# Viral CpG Deficiency Provides No Evidence That Dogs Were Intermediate Hosts for SARS-CoV-2

**DOI:** 10.1093/molbev/msaa178

**Published:** 2020-07-13

**Authors:** David D Pollock, Todd A Castoe, Blair W Perry, Spyros Lytras, Kristen J Wade, David L Robertson, Edward C Holmes, Maciej F Boni, Sergei L Kosakovsky Pond, Rhys Parry, Elizabeth J Carlton, James L N Wood, Pleuni S Pennings, Richard A Goldstein

**Affiliations:** m1 Department of Biochemistry & Molecular Genetics, University of Colorado School of Medicine, Aurora, CO; m2 Department of Biology, University of Texas Arlington, Arlington, TX; m3 MRC-University of Glasgow Centre for Virus Research (CVR), Glasgow, United Kingdom; m4 Marie Bashir Institute for Infectious Diseases & Biosecurity, School of Life & Environmental Sciences and School of Medical Sciences, The University of Sydney, Sydney, NSW, Australia; m5 5Center for Infectious Disease Dynamics, Department of Biology, Pennsylvania State University, University Park, PA; m6 Institute for Genomics and Evolutionary Medicine, Temple University, Philadelphia, PA; m7 Australian Infectious Disease Research Centre, School of Biological Sciences, The University of Queensland, Brisbane, QLD, Australia; m8 Department of Environmental and Occupational Health, Colorado School of Public Health, University of Colorado, Anschutz, Aurora, CO; m9 Disease Dynamics Unit, Department of Veterinary Medicine, University of Cambridge, Cambridge, United Kingdom; m10 Department of Biology, San Francisco State University, San Francisco, CA; m11 Division of Infection & Immunity, University College London, London, United Kingdom

**Keywords:** COVID-19, CpG deficiency, dogs, bats, pangolin

## Abstract

Due to the scope and impact of the COVID-19 pandemic there exists a strong desire to understand where the SARS-CoV-2 virus came from and how it jumped species boundaries to humans. Molecular evolutionary analyses can trace viral origins by establishing relatedness and divergence times of viruses and identifying past selective pressures. However, we must uphold rigorous standards of inference and interpretation on this topic because of the ramifications of being wrong. Here, we dispute the conclusions of Xia (2020. Extreme genomic CpG deficiency in SARS-CoV-2 and evasion of host antiviral defense. Mol Biol Evol. doi:10.1093/molbev/masa095) that dogs are a likely intermediate host of a SARS-CoV-2 ancestor. We highlight major flaws in Xia’s inference process and his analysis of CpG deficiencies, and conclude that there is no direct evidence for the role of dogs as intermediate hosts. Bats and pangolins currently have the greatest support as ancestral hosts of SARS-CoV-2, with the strong caveat that sampling of wildlife species for coronaviruses has been limited.

The COVID-19 pandemic began following a cross-species transmission event of the causative virus, SARS-CoV-2, sometime in late 2019 (Gorbalenya et al. 2020; Li et al. 2020; Lu et al. 2020; Zhang and Holmes 2020; Zhou, Yang, et al. 2020). As the scientific community works to understand the origins, biology, impacts, and treatment strategies for this virus, it is key that we avoid over interpretation of findings and speculation not well supported by available evidence. Otherwise, we risk diversion of time and resources from following more plausible and scientifically justified leads. Accordingly, there is a heightened urgency for the scientific community to diligently survey and critically evaluate new research findings before they are accepted as sound or actionable knowledge.

Understanding the prehuman origins of SARS-CoV-2 is important because it may provide insight into how and why it was able to jump into human populations, in turn better defining the risks of future pandemics. Molecular evolutionary studies have an important role to play in inferring the origins of the virus because they can confirm the relatedness of viruses, shed light on evolutionary time-scales, and potentially identify past selective pressures that allowed the virus to successfully infect and replicate in human hosts. A recent study by Xia (2020) used patterns of CpG deficiency in SARS-CoV-2 and related coronaviruses, and a series of compounding assumptions, to promote “the importance of monitoring SARS-like coronaviruses in feral dogs.” His conclusions rest upon the observation that values of CpG deficiency in SARS-CoV-2 (genus *Betacoronavirus*) resemble those observed in distantly related canine alphacoronaviruses that constitute a separate genus within the *Coronaviridae*. Here, we conduct a critical re-evaluation of the conclusions of Xia (2020), highlight key flaws in his underlying logic, and illustrate why his conclusion that dogs are likely intermediate hosts of SARS-CoV-2 is unjustified based on available data. We re-analyze viral CpG deficiency data to incorporate key pangolin viral genomes that were available but omitted from Xia’s study. These data further undermine the key inferences and conclusions of Xia (2020).

## Clarifying the Uncertainty in SARS-CoV-2 Origins

To date, the closest known relative of SARS-CoV-2 across its genome as a whole is the RaTG13 virus that was isolated from a horseshoe bat, the established reservoir of the earlier SARS coronaviruses that emerged in 2002–2003 (Zhou, Chen, et al. 2020). Interestingly, RmYN02, isolated from another horseshoe bat, is more closely related to SARS-CoV-2 in the long replicase 1a reading frame (orf1ab; Zhou, Yang, et al. 2020). The next closest relative of SARS-CoV-2, Pangolin-2019, was isolated from pangolins illegally smuggled into Guangdong province, China (Lam et al. 2020; Xiao et al. 2020). Thus, until a closer relative is identified, bats, followed by pangolins, are the most likely source of the originating or reservoir host species for SARS-CoV-2. However, all these viruses are divergent enough from SARS-CoV-2 on an evolutionary time-scale that their role is uncertain (Boni et al. 2020).

A potentially informative feature of the cluster of bat and pangolin coronaviruses similar to SARS-CoV-2 is a region of the Spike protein. This is a key viral feature that binds to the ACE2 receptor in SARS-CoV-2 to enter host cells, and shows strong signs of multiple past recombination events. The Spike binding regions of the Pangolin-2019 coronavirus, and that of the 2017 pangolin coronavirus sequence, are more similar to SARS-CoV-2 than that of RaTG13. This suggests that there were multiple recombination events between ancestral viruses related to the bat RaTG13, RmYN02, Pangolin-2019, and SARS-CoV-2 lineages (Boni et al. 2020). These findings suggest that such interviral recombination events occur commonly among coronaviruses in nature (Zhou, Chen, et al. 2020). Further, there was likely a recombination event in the past involving the variable loop region of the bat RaTG13 virus, although current sampling is insufficient to determine what the parental and offspring sequences were in this recombination event (Boni et al. 2020). For these recombination events to have occurred, divergent viruses must have co-infected the same host. Although bats are the only group known to host both ancestral forms of SARS-CoV-2, the two recent host-jumping events indicate that other organisms are also possible candidate hosts. The timing of these events is informed by the extent of divergence among these sequences and the viral mutation rate. Estimated divergence dates between SARS-CoV-2 and RaTG13, suggest that the coronavirus lineage that gave rise to SARS-CoV-2 circulated unnoticed for decades in bats or other intermediate hosts prior to infecting humans (Boni et al. 2020; Nielsen et al. 2020).

## Genomic Nucleotide Content Is Not Good Evidence to Implicate Viral Hosts

**Fig. 1:**
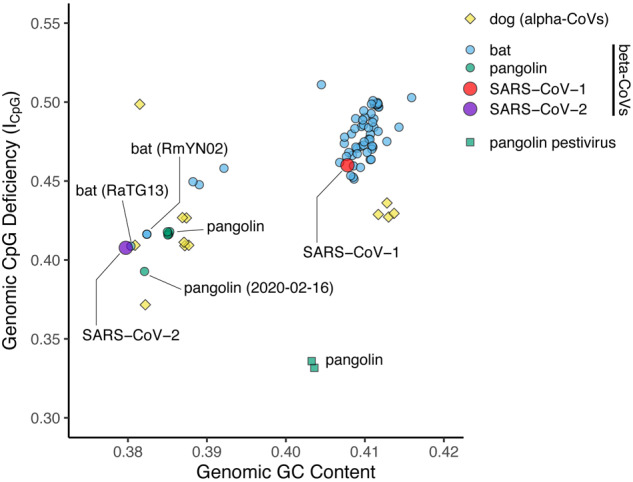
Coronavirus genomic CpG deficiency (*I*_CpG_) versus viral genomic GC content for select betacoronaviruses (beta-CoVs), and dog alphacoronaviruses (alpha-CoVs). Pangolin pestiviruses are also shown to illustrate variation in *I*_CpG_ in a single host.

A well-known feature of most RNA viruses is that they tend to have lower levels of CpG dinucleotides than expected based on the relative frequencies of C and G nucleotides independently (Karlin et al. 1994; Rima and McFerran 1997; Jenkins et al. 2001; Cheng et al. 2013). The SARS-CoV-2 viral genome is more depleted in CpGs than many related coronaviruses (fig. 1), a trait shared with distantly related alphacoronaviruses in dogs. Based primarily on this observation, Xia (2020) concluded that canines are a likely intermediate (prehuman) host for SARS-CoV-2. The idea is founded on the assumption that CpG levels in SARS-CoV-2 and dog alphacoronavirus are notably low, requiring an unusual environment to evolve, and that the gastro-intestinal tract of dogs is the singular prime candidate to provide that environment. However, the basis of this argument is undermined by the observation that the most closely related sequences from bats and pangolins, several of which were omitted from Xia’s (2020) analysis, are also highly depleted in CpGs (fig. 1 and supplementary table S1, Supplementary Material online). In addition, many other RNA viruses are far more depleted in CpGs than is SARS-CoV-2, including pestiviruses that also happen to be found in the pangolin (Gao et al. 2020; fig. 1). Hence, CpG depletion is not a unique feature of dog viruses or SARS-CoV-2.

Many factors can influence the genomic composition of viruses, including random genetic drift, recombination, and underlying stochastic mutational bias, as well as natural selection (Jenkins et al. 2001; Dunham et al. 2009; Theys et al. 2018). Normally in molecular evolutionary analyses, we assume mutation and drift as the null model, and inference of natural selection, adaptation, and recombination need to be demonstrated by obtaining strong evidence in their favor. Xia (2020), however, provided no compelling evidence for natural selection. It is reasonable to think that natural selection can play a role in viral CpG levels because viral CpG is a target for mammalian defense systems and viruses are likely to evolve to evade such host defense mechanisms. Nevertheless, the evolutionary reasons for low GC content are still debated in even exceptionally well-studied systems with unquestioned animal origins (e.g., HIV-1; Alinejad-Rokny et al. 2016; Antzin-Anduetza et al. 2017; Wasson et al. 2017). As Xia (2020) points out, the mammalian zinc finger antiviral protein (ZAP) binds to CpG dinucleotides in viral RNA genomes and inhibits viral replication and mediates viral degradation (Takata et al. 2017; Ficarelli et al. 2019; Meagher et al. 2019; FicarelLi et al. 2020). Additionally, mammalian APOBEC3G is known to modify viral RNA, deaminating C to U (Sharma et al. 2015, 2016, 2019). Notably, bats show unusual and extensive adaptation of APOBEC3G, potentially driving their antiviral response and perhaps correlating with low CpG content in SARS-like coronaviruses in bats (Jebb et al. 2020). At any point in time, natural selection affecting CpG content may be in a rough balance with mutation and drift, but differences in CpG content among species could be caused by strengthening or weakening of any of these factors. An altered host environment could induce more extensive targeting of CpGs and positive selection for their removal, or an altered viral life history could lead to stronger selection on viral protein function, including CpGs, and stronger selection for their retention. We can speculate that sequence context-dependency, such as that shown for GATC motifs (Hénaut et al. 1996), may also play a role. Likewise, relaxed selection could influence CpG levels in either direction. Further, it has been shown that the genomic dinucleotide composition of RNA viruses is a poor-predictor of host species, suggesting that there is minimal host-specific impact on CpG suppression (Di Giallonardo et al. 2017). For these reasons, gross similarities in CpG depletion characteristics are unreliable for inferring their shared causative nature.

In summary, CpG depletion levels are known to be diverse among RNA viruses broadly, CpG levels are also depleted in noncanine viruses closely related to SARS-CoV-2, evidence that natural selection drove the CpG depletion in SARS-CoV-2 ancestors is lacking, and there are a variety of competing mechanisms for genomes to become relatively depleted in CpG over evolutionary time. Despite this, Xia (2020) speculated that low viral genomic CpG levels in SARS-CoV-2 required evolutionary time in a previous host species and tissue that more actively selected for CpG depletion than do bats. Because low CpG levels, similar to those in SARS-CoV-2, were observed in alphacoronaviruses that infect dog digestive tracts, he then concluded: “… canine tissue infected by the canine coronavirus may provide a cellular environment selecting against CpG,” and “This suggests the importance of monitoring SARS-like coronaviruses in feral dogs in the fight against SARS-CoV-2.” However, there is no evidence for the logical premise of Xia’s argument, considering that all mammals have digestive tracts. Additionally, a recent inoculation study found that although other domesticated mammalian hosts are highly susceptible to SARS-CoV-2, canines exhibited low susceptibility, and no traces of viral RNA were detectable in any dog organs (Shi et al. 2020). Further, it is notable that based on a study modeling ACE2 binding affinity with the Spike protein from SARS-CoV-2, it seems highly unlikely that dogs played an important role in the recent evolution of SARS-Cov-2 (Damas et al. 2020). These findings cast further doubt on the relevance of dogs as hosts of viruses related to SARS-CoV-2. Hence, there is no reason to conclude that dogs or dog digestive tracts are special in this respect.

## Further Analysis Indicating That Viral CpG Depletion Levels Do Not Implicate Dogs

We reanalyzed the “SARS-related” subset of the data not only shown in figure 1 from Xia (2020), but also including seven betacoronaviruses from pangolins and a bat (RmYN02), four additional dog alphacoronaviruses, and two additional noncoronaviruses (pestiviruses) from pangolins, using the same indices (*I*_CpG_—a measure of genomic CpG deficiency, and genomic GC content; fig. 1). The names of all viruses used in our analysis, along with estimated GC content and *I*_CpG_ estimates, are provided in supplementary table S1, Supplementary Material online. Multiple bat and pangolin betacoronaviruses have low *I*_CpG_ comparable to SARS-CoV-2, and the other pangolin viruses have even lower *I*_CpG_. This nonexhaustive sample is sufficient to refute the claim by Xia (2020) that “no betacoronaviruses from their natural hosts have the genomic *I*_CpG_ and GC% combination close to SARS-CoV-2 and BatCoV RaTG13.” Notably, dog alphacoronaviruses are also not exceptional in terms of CpG deficiency. Furthermore, although humans and dogs have ZAP, which Xia (2020) hypothesizes targets and selects for CpG depletion, our analyses suggest ZAP is highly conserved in mammalian genomes. In particular, bat and pangolin genomes also appear to contain functional ZAP (supplementary table S2, Supplementary Material online). APOBEC3G may also be conserved across mammals, but the results are less clear, as similarity to human APOBEC3G is low in other mammals; however, human APOBEC3G is more similar to genes in bats and the pangolin than in dogs (supplementary table S3, Supplementary Material online). These results are relevant because they mean that bats and pangolins, the most likely prehuman hosts at present, have equal mechanistic potential to select against viral CpG content as dogs. Although there is no evidence that SARS-CoV-2 has a low CpG content due to the action or evasion of these mechanisms (or if such a process is responsible for any CpG patterns in any organisms), the distribution of these proteins provides no prior mechanistic basis to exclude bats and pangolins as either reservoirs or intermediate hosts, and provides no evidence to specifically implicate dogs.

**Fig. 2:**
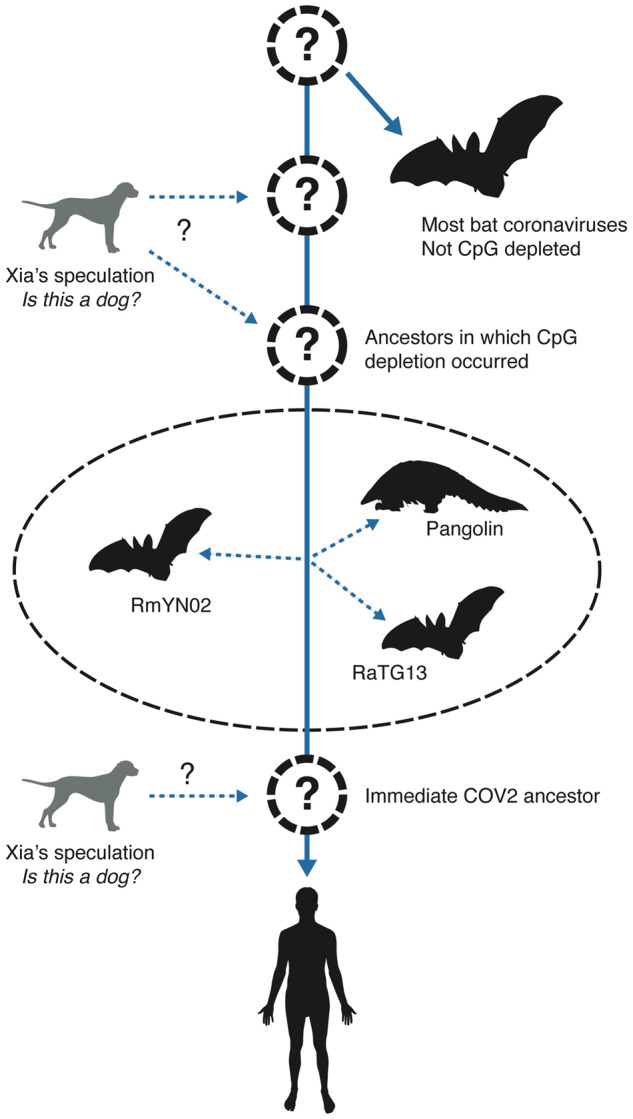
Prevailing origin and transmission hypotheses supported by recent literature. The organisms in black outline are host sources of viral sequences closely related to SARS-CoV-2. The dashed circles represent hosts carrying viruses on the ancestral lineage leading to SARS-Cov-2, with the large question marks indicating that despite the recurrence of bats as hosts of related viruses, the ancestral hosts are uncertain. Two ancestral hosts are indicated during the time of CpG depletion because this is a much longer timespan, and there could plausibly have been multiple hosts from divergent species during this time. Dogs are represented by gray outlines because no viruses closely related to SARS-CoV-2 have been discovered in dogs. Question mark labeled dashed arrows represent Xia’s (2020) dual speculations, that dogs may have been hosts during the process of CpG depletion and during recent ancestral SARS-CoV-2 evolution.

In addition to being unsupported by positive evidence, Xia’s (2020) hypothesis for dogs as intermediate hosts of ancestral viruses giving rise to SARS-CoV-2 requires an unlikely history of cross-species viral transmission (see fig. 2 for potential hypotheses) for which there is no evidence. Specifically, this hypothesis minimally requires: 1) an ancestral SARS virus in bats (the main reservoir for SARS-lineage viruses) was passed to dogs, which drove depletion of viral CpGs, 2) dogs passed this virus back to an unknown host or hosts that passed it to bats and pangolins (which gave rise to Pangolin2019, bat RmYN02, and bat RaTG13 observed coronaviruses), and 3) descendant lineages of this virus were passed to humans via an unknown host (fig. 2). In addition to this primary hypothesis, Xia’s manuscript and subsequent online comments further imply dogs were a more recent host of SARS-CoV-2, and thus the need for monitoring “in feral dogs” (fig. 2). A simpler alternative to this improbable transmission hypothesis is that bats transferred this virus directly to humans or through a yet undetermined host (fig. 2). In our view, it is a problem that potential wild animal hosts have not yet been well sampled. Although it may be worthwhile to test dog samples as part of broader efforts to sample diverse potential hosts, a narrow focus on dogs is unjustified by existing evidence.

In summary, the proposition of Xia (2020) that dogs are a likely prehuman host for SARS-CoV-2 is not justified by available evidence. Xia (2020) did not demonstrate that the low CpG frequency in the SARS-CoV-2 genome was driven by a unique selective environment in dog digestive tracts. The SARS-CoV-2 is also less virulent than other human betacoronaviruses (SARS-CoV-1 and MERS-CoV; Chen 2020; Munster et al. 2020), contradicting his assertion that CpG-deficient viruses are more virulent. Furthermore, closely related betacoronaviruses from bats and pangolins have CpG-deficiencies similar to SARS-CoV-2. Dogs are not more plausible than most other potential host species, and based on current data, far less plausible than bats or pangolins. Still, we are missing ∼20–70 years of the recent evolutionary history of the lineage leading to SARS-CoV-2, and we must broadly survey a wide range of wild and domestic species to uncover the origin of SARS-like coronaviruses.

## Supplementary Material

Supplementary data are available at *Molecular Biology and Evolution* online.

## Supplementary Material

msaa178_Supplementary_Data
