# Supplementary material for: Viral CpG Deficiency Provides No Evidence That Dogs Were Intermediate Hosts for SARS-CoV-2
Source: Mol Biol Evol. 2020 Jul 13;37(9):2706–10. doi: 10.1093/molbev/msaa178 (PMC7454803; doi:10.1093/molbev/msaa178)
Supplement: msaa178_Supplementary_Data [file msaa178_supplementary_data.zip › msaa178-Suppl_Data/SuppTables2-3_BlastResults_05.23.20.pdf]

Supplementary Table 2. Results of a BLASTp search of the human ZAP protein (NP_064504) against all *Manis* (pangolin), *Canis* (dog), and *Rhinolophus* (horseshoe bat) protein records on NCBI. The top hit in bats, pangolins, and dogs all exhibit comparable percent identity to the human ZAP protein.

ACCESSION	DESCRIPTION	COMMON IDENTIFIER	MAX SCORE	TOTAL SCORE	QUERY COVER	E VALUE	PER. IDENT
XP_032954599.1	zinc finger CCCH-type antiviral protein 1 isoform X1 [Rhinolophus ferrumequinum]	Horseshoe Bat	674	1177	99%	0	67.69%
XP_017536548.1	PREDICTED: zinc finger CCCH-type antiviral protein 1 isoform X3 [Manis javanica]	Pangolin	1226	1226	100%	0	66.04%
XP_032954601.1	zinc finger CCCH-type antiviral protein 1 isoform X2 [Rhinolophus ferrumequinum]	Horseshoe Bat	932	932	77%	0	65.62%
XP_017536547.1	PREDICTED: zinc finger CCCH-type antiviral protein 1 isoform X2 [Manis javanica]	Pangolin	1219	1219	100%	0	65.32%
XP_017536546.1	PREDICTED: zinc finger CCCH-type antiviral protein 1 isoform X1 [Manis javanica]	Pangolin	1215	1215	100%	0	64.75%
XP_005629620.1	zinc finger CCCH-type antiviral protein 1 [Canis lupus familiaris]	Dog	1170	1170	100%	0	64.64%
XP_005629621.1	zinc finger CCCH-type antiviral protein 1-like [Canis lupus familiaris]	Dog	105	105	11%	3.00E-25	46.30%
XP_025289633.1	zinc finger CCCH-type antiviral protein 1-like [Canis lupus dingo]	Dog	174	174	24%	2.00E-47	39.16%
XP_032954598.1	zinc finger CCCH-type antiviral protein 1-like [Rhinolophus ferrumequinum]	Horseshoe Bat	169	169	24%	6.00E-46	38.52%
XP_025289675.1	poly [ADP-ribose] polymerase 12 [Canis lupus dingo]	Dog	285	407	70%	2.00E-83	38.26%
XP_032955073.1	protein mono-ADP-ribosyltransferase PARP12 [Rhinolophus ferrumequinum]	Horseshoe Bat	291	411	68%	1.00E-85	37.64%
XP_017528692.1	PREDICTED: poly [ADP-ribose] polymerase 12 [Manis javanica]	Pangolin	266	361	62%	6.00E-77	36.21%
XP_025317656.1	poly [ADP-ribose] polymerase 11 isoform X3 [Canis lupus dingo]	Dog	121	121	18%	9.00E-30	33.50%
XP_032973803.1	protein mono-ADP-ribosyltransferase PARP11 isoform X2 [Rhinolophus ferrumequinum]	Horseshoe Bat	120	120	18%	2.00E-29	33.50%
XP_025330989.1	poly [ADP-ribose] polymerase 14 isoform X6 [Canis lupus dingo]	Dog	77.8	77.8	15%	2.00E-13	32.26%
XP_025330980.1	poly [ADP-ribose] polymerase 14 isoform X5 [Canis lupus dingo]	Dog	77.4	77.4	15%	2.00E-13	32.26%
XP_022269483.1	poly [ADP-ribose] polymerase 14 isoform X4 [Canis lupus familiaris]	Dog	77.4	77.4	15%	3.00E-13	32.26%
XP_025330958.1	poly [ADP-ribose] polymerase 14 isoform X2 [Canis lupus dingo]	Dog	92	92	19%	8.00E-18	31.63%
XP_025330953.1	poly [ADP-ribose] polymerase 14 isoform X1 [Canis lupus dingo]	Dog	91.7	91.7	19%	9.00E-18	31.63%
XP_850880.2	poly [ADP-ribose] polymerase 14 isoform X5 [Canis lupus familiaris]	Dog	91.7	91.7	19%	1.00E-17	31.63%
XP_022269481.1	poly [ADP-ribose] polymerase 14 isoform X2 [Canis lupus familiaris]	Dog	91.7	91.7	19%	1.00E-17	31.63%
XP_005639627.1	poly [ADP-ribose] polymerase 14 isoform X1 [Canis lupus familiaris]	Dog	91.3	91.3	19%	1.00E-17	31.63%
XP_032987823.1	protein mono-ADP-ribosyltransferase PARP14 [Rhinolophus ferrumequinum]	Horseshoe Bat	82	82	19%	8.00E-15	31.47%
XP_032987630.1	protein mono-ADP-ribosyltransferase PARP15 isoform X5 [Rhinolophus ferrumequinum]	Horseshoe Bat	86.3	86.3	17%	2.00E-16	30.81%
XP_032987586.1	protein mono-ADP-ribosyltransferase PARP15 isoform X1 [Rhinolophus ferrumequinum]	Horseshoe Bat	86.3	86.3	17%	2.00E-16	30.81%
XP_032987602.1	protein mono-ADP-ribosyltransferase PARP15 isoform X3 [Rhinolophus ferrumequinum]	Horseshoe Bat	86.3	86.3	17%	2.00E-16	30.81%
XP_032987613.1	protein mono-ADP-ribosyltransferase PARP15 isoform X4 [Rhinolophus ferrumequinum]	Horseshoe Bat	85.9	85.9	17%	3.00E-16	30.81%
XP_022269542.1	poly [ADP-ribose] polymerase 15 isoform X4 [Canis lupus familiaris]	Dog	85.5	85.5	18%	4.00E-16	30.81%
XP_025331073.1	poly [ADP-ribose] polymerase 15 isoform X4 [Canis lupus dingo]	Dog	85.5	85.5	18%	4.00E-16	30.81%
XP_005639622.1	poly [ADP-ribose] polymerase 15 isoform X1 [Canis lupus familiaris]	Dog	85.1	85.1	18%	7.00E-16	30.81%
XP_005639625.1	poly [ADP-ribose] polymerase 15 isoform X3 [Canis lupus familiaris]	Dog	84.7	84.7	18%	7.00E-16	30.81%
XP_022269540.1	poly [ADP-ribose] polymerase 15 isoform X2 [Canis lupus familiaris]	Dog	84.7	84.7	18%	7.00E-16	30.81%
XP_025331009.1	poly [ADP-ribose] polymerase 15 isoform X1 [Canis lupus dingo]	Dog	84.7	84.7	18%	8.00E-16	30.81%
XP_025331054.1	poly [ADP-ribose] polymerase 15 isoform X3 [Canis lupus dingo]	Dog	84.7	84.7	18%	8.00E-16	30.81%
XP_025331045.1	poly [ADP-ribose] polymerase 15 isoform X2 [Canis lupus dingo]	Dog	84.7	84.7	18%	9.00E-16	30.81%
XP_017505555.1	PREDICTED: TCDD-inducible poly [ADP-ribose] polymerase [Manis javanica]	Pangolin	139	139	42%	5.00E-33	28.90%
XP_025291904.1	TCDD-inducible poly [ADP-ribose] polymerase [Canis lupus dingo]	Dog	137	137	42%	2.00E-32	28.54%
XP_032988082.1	protein mono-ADP-ribosyltransferase TIPARP [Rhinolophus ferrumequinum]	Horseshoe Bat	138	138	42%	8.00E-33	28.44%
XP_013964023.1	poly [ADP-ribose] polymerase 11 isoform X2 [Canis lupus familiaris]	Dog	142	142	32%	2.00E-36	28.40%
XP_025317652.1	poly [ADP-ribose] polymerase 11 isoform X1 [Canis lupus dingo]	Dog	142	142	32%	4.00E-36	28.40%
XP_017531639.1	PREDICTED: poly [ADP-ribose] polymerase 14 [Manis javanica]	Pangolin	80.5	80.5	18%	2.00E-14	28.19%
XP_032973799.1	protein mono-ADP-ribosyltransferase PARP11 isoform X1 [Rhinolophus ferrumequinum]	Horseshoe Bat	140	140	32%	1.00E-35	28.09%
XP_017525977.1	PREDICTED: LOW QUALITY PROTEIN: poly [ADP-ribose] polymerase 11 [Manis javanica]	Pangolin	134	134	32%	3.00E-33	27.01%
XP_032982126.1	protein mono-ADP-ribosyltransferase PARP10 isoform X2 [Rhinolophus ferrumequinum]	Horseshoe Bat	68.2	68.2	16%	1.00E-10	25.31%
XP_032982124.1	protein mono-ADP-ribosyltransferase PARP10 isoform X1 [Rhinolophus ferrumequinum]	Horseshoe Bat	68.2	68.2	16%	1.00E-10	25.31%

Supplementary Table 3. Results of a BLASTp search of the human APOBEC-3G protein (NP_068594) against all *Manis* (pangolin), *Canis* (dog), and *Rhinolophus* (horseshoe bat) protein records on NCBI. The top hit in bats, pangolins, and dogs all exhibit comparable percent identity to the human APOBEC-3G protein.

ACCESSION	DESCRIPTION	COMMON IDENTIFIER	MAX SCORE	TOTAL SCORE	QUERY COVER	E VALUE	PER. IDENT
XP_032972590.1	DNA dC->dU-editing enzyme APOBEC-3A-like [Rhinolophus ferrumequinum]	Horseshoe Bat	191	281	96%	2.00E-58	52.13%
XP_032974400.1	DNA dC->dU-editing enzyme APOBEC-3G-like [Rhinolophus ferrumequinum]	Horseshoe Bat	189	273	96%	3.00E-58	51.85%
XP_017499606.1	PREDICTED: DNA dC->dU-editing enzyme APOBEC-3A [Manis javanica]	Pangolin	164	247	96%	3.00E-48	50.79%
DAA05239.2	TPA: activation-induced cytidine deaminase [Canis lupus familiaris]	Dog	41.2	41.2	9%	2.00E-04	48.57%
XP_025327527.1	DNA dC->dU-editing enzyme APOBEC-3A-like [Canis lupus dingo]	Dog	165	242	95%	1.00E-47	48.13%
DAA80475.1	TPA: activation-induced cytidine deaminase [Canis lupus familiaris]	Dog	115	193	66%	4.00E-30	47.66%
NP_001333061.1	apolipoprotein B mRNA editing enzyme, catalytic polypeptide-like 3Z1 [Canis lupus familiaris]	Dog	166	244	95%	4.00E-49	47.62%
XP_017515433.1	PREDICTED: single-stranded DNA cytosine deaminase [Manis javanica]	Pangolin	148	238	95%	6.00E-42	45.41%
XP_032973610.1	single-stranded DNA cytosine deaminase [Rhinolophus ferrumequinum]	Horseshoe Bat	147	235	95%	2.00E-41	44.86%
NP_001003380.1	single-stranded DNA cytosine deaminase [Canis lupus familiaris]	Dog	143	230	95%	4.00E-40	44.32%
XP_022266563.1	single-stranded DNA cytosine deaminase isoform X1 [Canis lupus familiaris]	Dog	144	231	95%	3.00E-40	42.71%
NP_001333059.1	apolipoprotein B mRNA editing enzyme, catalytic polypeptide-like 3Z3 [Canis lupus familiaris]	Dog	134	232	86%	1.00E-36	40.51%
XP_025327514.1	DNA dC->dU-editing enzyme APOBEC-3G-like [Canis lupus dingo]	Dog	245	476	99%	2.00E-74	37.15%
XP_032973765.1	C->U-editing enzyme APOBEC-1 [Rhinolophus ferrumequinum]	Horseshoe Bat	74.3	140	48%	2.00E-14	35.96%
XP_032972588.1	LOW QUALITY PROTEIN: DNA dC->dU-editing enzyme APOBEC-3F-like [Rhinolophus ferrumequinum]	Horseshoe Bat	234	591	98%	3.00E-70	35.64%
XP_025327805.1	C->U-editing enzyme APOBEC-1 [Canis lupus dingo]	Dog	79	149	51%	6.00E-16	33.06%
XP_005637246.1	C->U-editing enzyme APOBEC-1 [Canis lupus familiaris]	Dog	79	149	51%	7.00E-16	33.06%
XP_032951269.1	C->U-editing enzyme APOBEC-2 [Rhinolophus ferrumequinum]	Horseshoe Bat	83.2	83.2	48%	2.00E-17	29.84%
XP_017535100.1	PREDICTED: C->U-editing enzyme APOBEC-1 [Manis javanica]	Pangolin	68.6	134	53%	2.00E-12	28.89%
XP_538909.2	C->U-editing enzyme APOBEC-2 [Canis lupus familiaris]	Dog	78.6	78.6	48%	7.00E-16	28.79%
XP_025274491.1	C->U-editing enzyme APOBEC-2 [Canis lupus dingo]	Dog	77.8	77.8	48%	1.00E-15	28.79%
XP_017508596.1	PREDICTED: C->U-editing enzyme APOBEC-2 [Manis javanica]	Pangolin	74.3	74.3	48%	2.00E-14	28.50%
